# COVID-19 in Malaysia: Descriptive Epidemiologic Characteristics of the First Wave

**DOI:** 10.3390/ijerph19073828

**Published:** 2022-03-23

**Authors:** Sumarni Mohd Ghazali, Sarbhan Singh, Asrul Anuar Zulkifli, Yoon Ling Cheong, Nuur Hafizah Md Iderus, Ahmed Syahmi Syafiq Md Zamri, Nadhar Ahmad Jaafar, Chee Herng Lai, Wan Noraini Wan Mohamed Noor, Norhayati Rusli, Chee Kheong Chong, Tahir Aris, Hishamshah Mohd Ibrahim, Sarat Chandra Dass, Balvinder Singh Gill

**Affiliations:** 1 Institute for Medical Research, Ministry of Health, Shah Alam 40170, Malaysia; sumarni.mg@moh.gov.my (S.M.G.); issarbhan@moh.gov.my (S.S.); cheongyl@moh.gov.my (Y.L.C.); nuurhafizah@moh.gov.my (N.H.M.I.); syahmi.syafiq@moh.gov.my (A.S.S.M.Z.); nadhar@moh.gov.my (N.A.J.); joshua0505@gmail.com (C.H.L.); tahir.a@moh.gov.my (T.A.); drbsgill@moh.gov.my (B.S.G.); 2 Ministry of Health, Putrajaya 62590, Malaysia; drwnoraini@moh.gov.my (W.N.W.M.N.); dr_norhayati@moh.gov.my (N.R.); drchongck@moh.gov.my (C.K.C.); drhishamshah@moh.gov.my (H.M.I.); 3 School of Mathematical and Computer Sciences, Heriot-Watt University, Putrajaya 62200, Malaysia; s.dass@hw.ac.uk

**Keywords:** COVID-19, epidemiology, disease transmission, first wave

## Abstract

This study aimed to describe the characteristics of COVID-19 cases and close contacts during the first wave of COVID-19 in Malaysia (23 January 2020 to 26 February 2020), and to analyse the reasons why the outbreak did not continue to spread and lessons that can be learnt from this experience. Characteristics of the cases and close contacts, spatial spread, epidemiological link, and timeline of the cases were examined. An extended SEIR model was developed using several parameters such as the average number of contacts per day per case, the proportion of close contact traced per day and the mean daily rate at which infectious cases are isolated to determine the basic reproduction number (R_0_) and trajectory of cases. During the first wave, a total of 22 cases with 368 close contacts were traced, identified, tested, quarantine and isolated. Due to the effective and robust outbreak control measures put in place such as early case detection, active screening, extensive contact tracing, testing and prompt isolation/quarantine, the outbreak was successfully contained and controlled. The SEIR model estimated the R_0_ at 0.9 which further supports the decreasing disease dynamics and early termination of the outbreak. As a result, there was a 11-day gap (free of cases) between the first and second wave which indicates that the first wave was not linked to the second wave.

## 1. Introduction

On 30 January 2020, the World Health Organization (WHO) announced an outbreak of a novel coronavirus in China as a Public Health Emergency of International Concern (PHEIC) on 30 January 2020, and a pandemic on 11 March 2020 [1]. The virus was subsequently named SARS-CoV-2, and the disease it causes, COVID-19. SARS-CoV-2 is a positive-sense single-stranded RNA virus belonging to the Coronaviridae family [2] similar to the severe acute respiratory syndrome coronavirus (SARS-CoV-1) and Middle East Respiratory Syndrome coronavirus (MERS-CoV), both of which have caused global outbreaks, SARS-CoV-1 in 2003 [3] and MERS-CoV in 2012 [4]. As of June 2021, more than 179 million people have been infected and more than 3.9 million have died from COVID-19, worldwide [5]. Epidemiological description and case characterizations are vital for a targeted outbreak response. As the COVID-19 infection is a novel pathogen, studies assessing the epidemiology of COVID-19 in different settings are required to further strengthen our understanding of the disease [6].

Malaysia is a country in South East Asia with a population of over 32 million in 2020. The first COVID-19 case in Malaysia was detected on 25 January 2020, and 21 more were reported in the following days. After 15 February 2020, no new cases were reported during the subsequent 11 consecutive days, and cases during the second wave, which started on 27 February 2020 were unrelated and had no epidemiological link to any of the first 22 cases. Thus, these 22 cases were regarded as the first wave, and later cases were categorized as the second COVID-19 wave of infections. Therefore, the first wave was an isolated event because there was no epidemiological link between the first wave and second wave, as evidenced by the 11 days’ gap of no cases between the two waves and the variations in the factors that resulted in these waves, wherein the first wave was due to importation of the virus into Malaysia by travellers from other countries (i.e., China, Singapore) whereas the second wave was due to mass gathering events [7,8]. With Malaysia experiencing several waves of COVID-19 [9], there is a need to analyse the data in the first wave to better understand its dynamics, which subsequently resulted in the ending of that cluster of cases.

Hence, this study aims to describe the characteristics of COVID-19 cases and close contacts during the first wave of COVID-19 in Malaysia (23 January 2020 to 26 February 2020), to analyse the reasons why the outbreak did not continue to spread and lessons that can be learnt from this experience. This is necessary to obtained better understanding regarding the epidemiology of the COVID-19 outbreak in Malaysia during the initial stages when there was no effect of movement restriction, vaccination, and institution of public health social measures (PHSM). Information on the initial epidemiological characteristic of COVID-19 could be of assistance in the management of the initial stages of future pandemics of novel infectious diseases.

## 2. Materials and Methods

We conducted a retrospective descriptive study of cases during the first COVID-19 wave in Malaysia. In addition, we modelled the first wave to determine its transmissibility and outbreak progression. Data on COVID-19 positive cases and close contacts from 23 January 2020 to 26 February 2020 were obtained from the Crisis Response and Preparedness Centre (CPRC), Ministry of Health Malaysia (MOH). The COVID-19 case data consisted of demographics (gender, age, nationality, type of case, source of detection, symptoms, comorbidity), clinical and admission data (date of onset of symptoms, date of admission, admitting hospital, case severity, type of treatment, dates of test results, patient outcome) and travel history (mode of travel, point of entry, screening type, location of screening, point of detection).

We described the socio-demographic data (gender, age, nationality, type of contact) of the close contacts obtained from contact tracing. The definition of close contacts, according to the Ministry of Health Malaysia guidelines (Guideline No. 3/2020) [10], were persons with healthcare associated exposure without appropriate personal protective equipment (PPE), which included providing direct care for COVID-19 patients, worked with health care workers infected with COVID-19, visited, or stayed in the same closed environment as a COVID-19 patient. This also includes people who worked together in close proximity or shared the same classroom environment with a COVID-19 patient, travelled together with a COVID-19 patient in any kind of conveyance or lived in the same household as a COVID-19 patient. 

Characteristics of cases and contacts are described in percentages, frequencies, means and standard deviations where appropriate and the epidemiological links between clusters of cases were described. The travel routes and points of entry into Malaysia for imported COVID-19 cases were illustrated visually on a map. Timelines for arrival in Malaysia, onset of symptoms, hospital admission/isolation, laboratory confirmation and discharge for each case were described in a timeline diagram.

We applied an extended Susceptible-Exposed-Infectious-Removed (SEIR) model using COVID-19 case data to determine the basic reproduction number and trajectory of cases during the first wave. The model was derived according to the method published by Gill et al. [11] with some modifications, using ODIN, an online disease modelling interface developed by Imperial College London [12]. The model used daily COVID-19 case data from the first wave, sourced from the Ministry of Health Malaysia official website [13]. The extended SEIR model was fitted using cases observed from 25 January 2020 to 15 February 2020. The model parameters are as shown in Table 1. A more detailed description of the model formulation and validation has been published by Gill et al. [11].

## 3. Results

### 3.1. Characteristics of COVID-19 Cases in the First Wave

**Figure 1:**
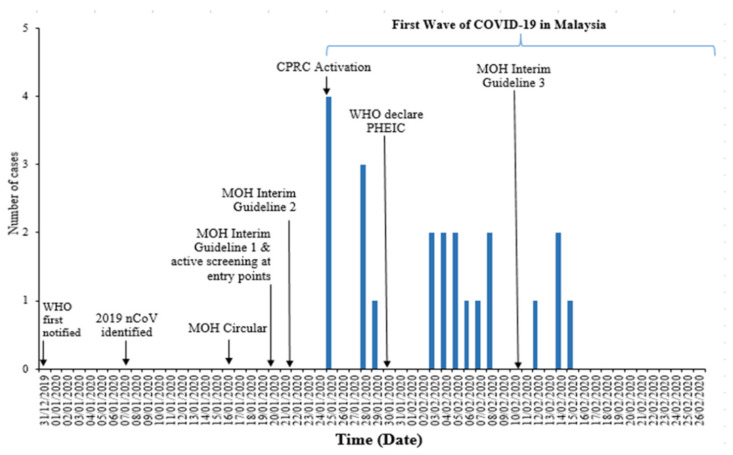
Epidemic curve of first wave of COVID-19 in Malaysia.

A total of 22 COVID-19 cases were reported in Malaysia during the first wave (Figure 1). Twenty cases (90.9%) were imported, 19 of which had recently come from China or its territories, and only two cases were locally transmitted with no recent history of international travel. The majority of the imported cases were Chinese nationals (*n* = 15, 68.2%) followed by Malaysians (*n* = 6, 27.3%) and one American (passenger of the MS Westerdam cruise ship returning from Cambodia to the United States via Kuala Lumpur International Airport (KLIA)). More than half of the cases were female (54.5%) with a mean age of 40.7 (SD = 21.6), and all cases were admitted and isolated in eight hospitals (Table 2).

**Figure 2:**
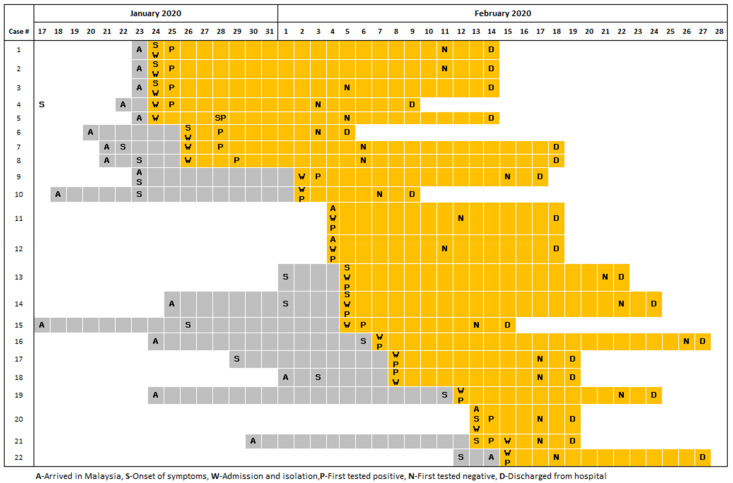
Timelines of the 22 cases in the first wave of COVID-19 infections in Malaysia.

All but one case (case #17) had no comorbidities. Almost all were symptomatic (90.9%), with fever (85%) and cough (70%) being the most predominant symptoms (Table 2). Among the 20 imported cases, only four (20%) were symptomatic upon arrival in Malaysia, 14 (70%) developed symptoms subsequently, and two (cases #11 and #12) were asymptomatic throughout, from arrival in Malaysia until they were discharged from isolation (14 days after testing positive). The duration from arrival to admission ranged from 0–19 days, with a mean of 6.8 days (SD = 6.8). The mean duration from arrival to admission among symptomatic vs. asymptomatic cases at point of arrival was 3.3 days and 7.6 days, respectively. Mean duration of hospitalization was 14.9 days and ranged from 4–23 days. By 27 February 2020, all 22 cases had recovered and were discharged, and there were no deaths reported during the first wave (Figure 2).

### 3.2. Spatial Spread

**Figure 3:**
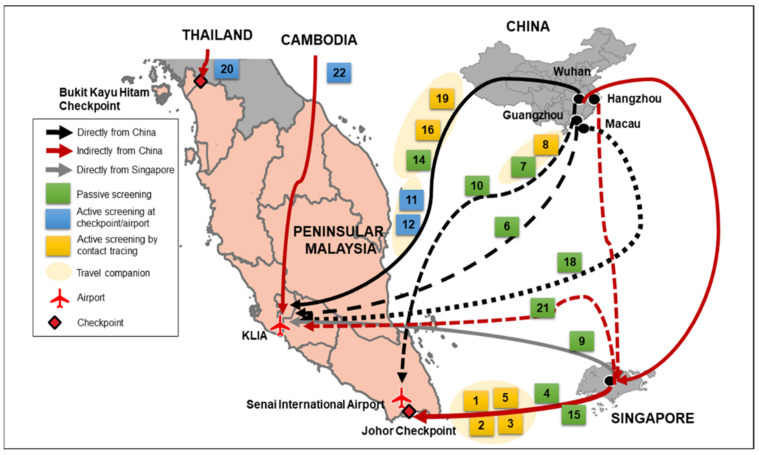
Spatial spread of COVID-19 into Malaysia during the first wave. (Numbers in boxes represent the case number).

A total of 13 (65%) imported cases came to Malaysia by air travel, while the remaining (35%) entered Malaysia by land. Among the 20 cases, 10 came directly from China (Wuhan, *n* = 8; Guangzhou, *n* = 1; and Macau, *n* = 1) wherein among them, seven cases arrived at KLIA and three cases at Senai airport in Johor. The remaining nine imported cases had travelled indirectly from China to Malaysia, transiting in other countries before arriving in Malaysia, as the following: seven cases via Singapore (six by bus, and one by flight that landed at KLIA) and one via Cambodia (flight landed at KLIA) and one via Thailand through the Bukit Kayu Hitam border checkpoint. One case was imported directly from Singapore (landed at KLIA). More than half (59.1%) of the cases were detected through active screening, and 40.9% through passive screening. Among the 20 imported cases, only four cases were detected through point of entry screening (cases #11, #12, #20 and #22). The other seven cases were detected by contact tracing and nine by passive screening (Figure 3).

### 3.3. Estimation of Reproduction Number

**Figure 4:**
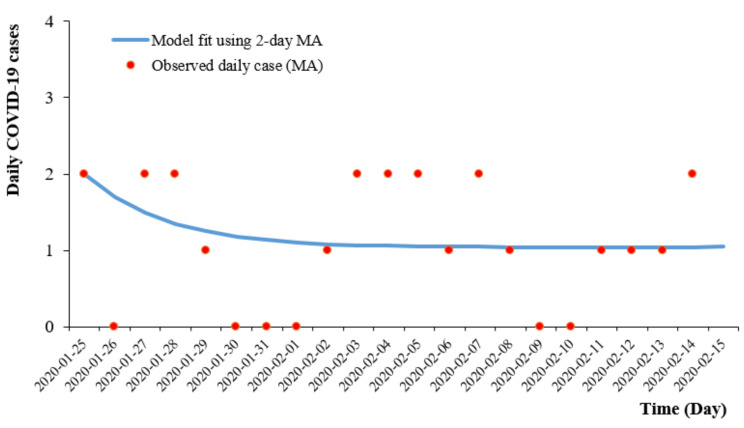
SEIR model fit of first wave COVID-19 cases in Malaysia.

Our model calibration estimated that the death rate due to COVID-19 (ε), the average number of contacts per day per case (ζ) and the mean daily rate at which infectious cases are isolated (δ) were 0, 4.82 and 0.30, respectively. Following model calibration, the best model fit estimated the basic reproduction number at 0.9. The SEIR model developed using parameters from cases during the first wave showed a decaying trend and predicted the end of the outbreak by mid-February 2020 as shown in Figure 4.

### 3.4. Epidemiological Link

Epidemiological links were found for 63.6% (*n* = 14) of the cases and five clusters were identified as follows:

*Cluster 1*

On 23 January 2020, Singaporean authorities notified the Malaysian government regarding eight members of a family who were close contacts of their first COVID-19 case (a 66-year-old male from Wuhan, China) who had travelled from Singapore to Johor Baharu. The close contacts were traced and four of them were subsequently diagnosed with COVID-19 (Cases #1, #2, #3 and #5).

*Cluster 2*

Cases #7 and #8 were a husband-and-wife couple travelling from Wuhan, China. The husband was first detected positive.

*Cluster 3*

Case #9 was a Malaysian businessman who had returned from a work-related trip to Singapore in January 2020 and travelled to his hometown in Kedah to celebrate the Chinese lunar new year, and then returned to Kuala Lumpur where he was diagnosed COVID-19 positive. Subsequently, two of his close contacts also tested positive, cases #13 and #17. The latter two cases were the first identified cases of COVID-19 local transmission in the country.

*Cluster 4*

A Malaysian father and his son, who were flown out of Wuhan, China back to Malaysia on one of several government-chartered flights to evacuate Malaysian citizens (Case #11 and #12).

*Cluster 5*

Case #14 was a 37-year-old Chinese national who had two friends travelling with her who eventually tested positive (Case #16 and #19). 

**Figure 5:**
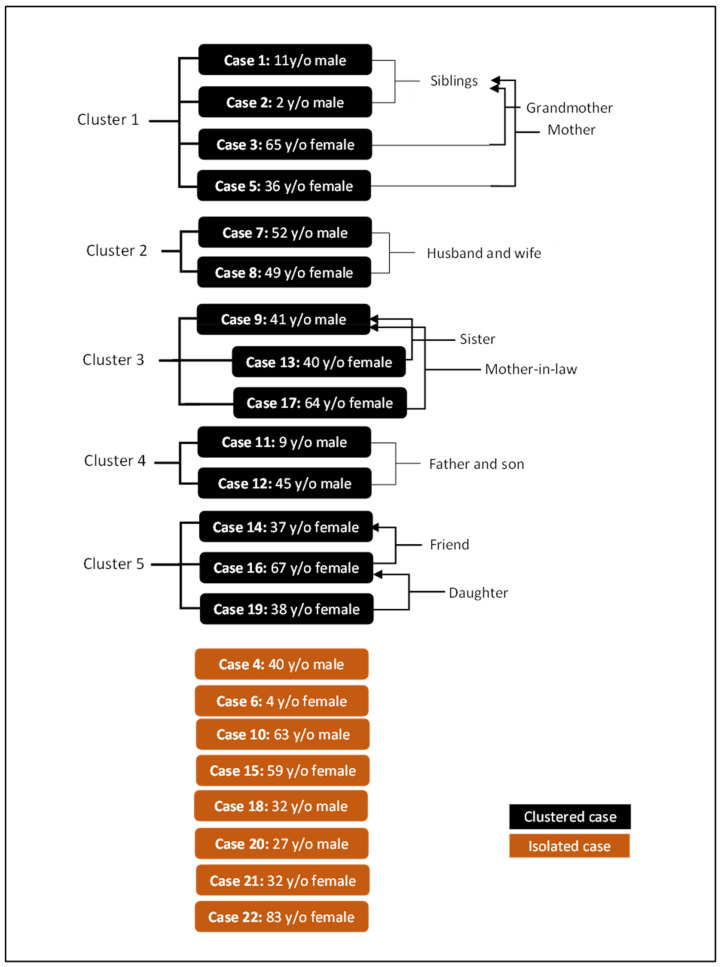
Epidemiological linkages among the first 22 COVID-19 cases in Malaysia.

These clusters are illustrated in Figure 5. Eight cases appeared to be isolated with no apparent link with any of the other cases. A majority of the isolated cases were Chinese nationals (*n* = 6).

### 3.5. Characteristics of Close Contacts

**Figure 6:**
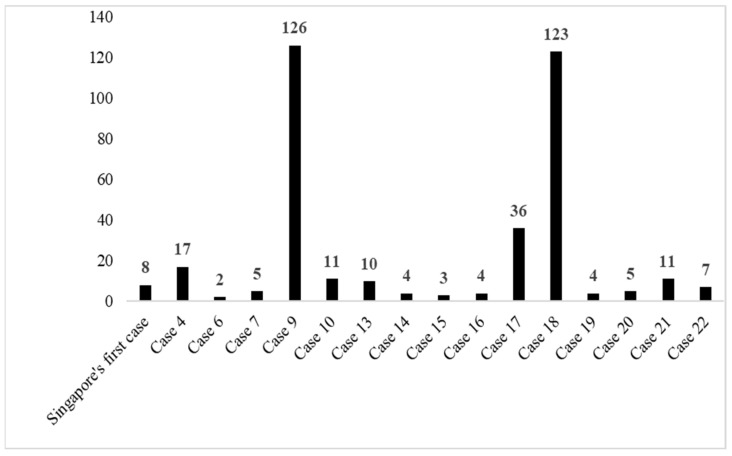
Number of close contacts traced by case in the first wave of COVID-19 in Malaysia.

There was a total of 368 unique close contacts after removing shared contacts, with an average of 16.6 contacts per case (Figure 6). Case #9 had the highest number of close contacts (*n* = 126), while three cases had none (case #8, #11 and #12). Case #8 had only one close contact which was her husband, who had been diagnosed before her (case #7). Case 11 and 12 had no contacts as they were among Malaysians evacuated from Wuhan by a special chartered flight, and all the passengers on the flight were classified as Persons Under Investigation (PUI) and screened upon arrival. A majority of the close contacts were female (57.4%), the mean age was 34.9 years and 78.3% were Malaysian citizens. Healthcare workers comprised 29.3% of the contacts, contacts on flight (26.0%), family (14.6%) and work colleagues (1.9%), and other types of contacts (28.2%) (Table 3). Nine of the close contacts subsequently tested positive (cases #1, #2, #3, #5, #8, #13, #16, #17 and #19).

## 4. Discussion

In this study, we described the characteristics of COVID-19 cases and close contacts during the first wave in Malaysia and their relation to entry point screening, international travel restrictions, contact tracing, isolation, and quarantine measures. In addition, we discuss possible explanations for why the first wave of COVID-19 in Malaysia subsequently ended. 

Most of the cases during the first wave of COVID-19 in Malaysia were equally distributed across both genders and were predominantly adults aged 19 years and above. Early studies from China [17,18,19], and Malta [20] reported higher proportions of males among their COVID-19 cases. Regarding age distribution, similar findings were reported in previous studies [17,19,20,21]. This finding can be attributed to higher risk of acquiring infection among adults in this age group due to increased exposure at work or travelling to work as well as underlying co-morbidities [19]. However, it was too early and the numbers too small to establish gender predisposition in COVID-19 infections.

The majority of COVID-19 cases during the first wave were imported cases from China, who travelled to Malaysia via air either directly or indirectly. Upon arrival into Malaysia, only a small proportion of these infected individuals were successfully detected through active screening at the point of entry. Two asymptomatic cases were detected only through targeted testing of Malaysians evacuated from Wuhan. This suggests that points of entry screening were unable to detect the majority of COVID-19 cases entering Malaysia. There could be several reasons for this. The nature of screening at entry points that rely mainly on thermal screening and self-health declaration are subject to many limitations. Firstly, individuals who are infected, but remain asymptomatic throughout the disease progression would not be routinely detected via screening at entry points, as in this study, two cases remained asymptomatic throughout. Likewise, those who are incubating the disease and thus pre-symptomatic at arrival or symptomatic but afebrile and provide inaccurate self-health declarations would also not be detected [22]. A study estimated 46% (95% CI: 36 to 58%) of infected travellers would not be detected, depending on incubation period, sensitivity of exit and entry screening, and due to asymptomatic status of the disease [23]. Therefore, thermal screening and self-health declaration at entry points is useful, but has limited effectiveness in preventing entry of the diseased individuals into the country. 

Quarantine of suspected cases and isolation of confirmed cases are two important strategies for disease containment. In the first wave, cases that were symptomatic at point of arrival in Malaysia were isolated earlier compared to asymptomatic cases (mean of 3.3 days vs. 7.6 days after arrival). This finding is reassuring, as the risk of disease transmission is reduced due to the early isolation of infected individuals. Similar findings have been reported in China whereby cases were admitted within 2 days after onset of symptoms [24,25]. Among the reasons contributing to early isolation of symptomatic individuals is the presence of symptoms among these individuals that increase their chances of being detected upon screening at entry points. Moreover, symptomatic individuals are more likely to seek treatment from health facilities, therefore increasing their chances of being detected [24]. Asymptomatic individuals were isolated one week on average after arrival in Malaysia. This is concerning, as there is a possibility of ongoing disease transmission by these asymptomatic individuals prior to being isolated. Therefore, one of the ways to enhance the detection of asymptomatic individuals is through a comprehensive and effective contact tracing system.

In this study, a total of 343 close contacts were identified, traced, tested, isolated and strictly quarantine for 20 of the COVID-19 cases. From this finding, it is evident that extensive contact tracing was conducted during the first wave of COVID-19 in Malaysia. As a result, 9 cases were identified, and five clusters were detected from contact tracing. This confirms the presence of epidemiological links among the cases during the first wave of COVID-19 in Malaysia. In addition, following the comprehensive contact tracing performed, asymptomatic and symptomatic individuals were detected and isolated early on, therefore, preventing community transmission. Thus, this indicates that extensive contact tracing is an important measure that would address the shortcomings of entry point screening. However, a study of COVID-19 contact tracing in Taiwan reported a low transmission rate of COVID-19 in their cohort and suggested that most transmissions occur very early in the disease, even before symptoms manifest, such that probability of infection of contacts decreases over time [26]. 

Clustering of cases were similarly reported in China [18,25,27], South Korea [28], Singapore [29] and Japan [30]. As transmission of COVID-19 is mainly through respiratory droplets, individuals in close proximity have increased chances of getting infected. This highlights the importance of early contact tracing in order to detect more potential cases and enable immediate isolation of the positive ones. Suspected cases or PUIs who are not admitted should be home quarantined until declared fit for release. Therefore, in order to break and eventually prevent sustained local transmission, it is crucial that the COVID-19 clusters be identified early on so that effective preventive and control measures can be initiated in a timely manner. There were eight cases with no clear epidemiological links, as these cases acquired the infection outside Malaysia and therefore establishing an epidemiological link was not possible due to international constraints. This is because all eight were imported cases, hence links to these cases could not be established as they were infected by a source outside of Malaysia, and prompt isolation prevented further transmission. Had there been isolated cases of probable local transmission, this would cause more alarm, as it would suggest on-going local transmission. In view of contact tracing pointing to sources outside Malaysia and the limitations of entry point screening, there is a need to stem importation of cases.

In order to limit further importation of COVID-19 cases into Malaysia, international travel restrictions were imposed beginning 18 March 2020, 50 days after the first COVID-19 cases was detected in Malaysia. This was in addition to the 14-day self-quarantine measure that had already been imposed on travellers entering Malaysia beginning from 4 January 2020 [9]. The city of Wuhan in China, the initial epicentre of the COVID-19 outbreak was placed under lockdown just 3 days after official confirmation of human-to-human transmission [31]. Singapore began barring short-term visitors from entering and transiting in the country on 22 March 2020 [32] and Thailand on 26 March 2020 [33]. Chinazzi et al. [34] ran simulations to model the international spread of COVID-19 using the global epidemic and mobility model (GLEAM) which showed international travel restriction would reduce importation of cases initially, however the effect would not be sustained unless coupled with other public health and behavioural interventions, which proved to be true. 

With entry point screening, quarantine and contact tracing in place, the transmission dynamics during the first wave of COVID-19 did not allow for the outbreak to be self-sustaining due to the small number of cases and effective early containment and control measures. Furthermore, most were imported cases which were identified and isolated early, thereby reducing the risk of community transmission. This was established by the extended SEIR model, wherein the outbreak parameters for the first wave model determined an R value of 0.9 which resulted in model fit and case trajectory that showed a quick decay in cases and cessation of the outbreak. Wherein during the first wave, cases were only observed for 22 days, following which the outbreak ended and no cases were reported subsequently for 11 days. Similarly, during the severe acute respiratory syndrome (SARS) outbreak from 2002 to 2003, Malaysia reported a small number of cases (*n* = 5) compared to other countries (i.e., Canada, China, Singapore) [35] and this was attributed to the institution of similar robust effective control measures as in the first wave of COVID-19 [36]. 

However, measures such as active screening, extensive contact tracing and prompt isolation/quarantine may not be as effective in subsequent waves due to larger case numbers, overwhelmed health care systems and variation in outbreaks propagation factors (i.e., mass gathering events and the introduction of new variants of concern.) [7,8,11]. The case numbers during the first wave were much lower compared to the second (*n* = 10,125) and third (*n* = 2,751,305). Similarly, the first wave lasted for a shorter duration compared to the second (*n* = 206 days) and third (*n* = 468 days) [37]. The first wave occurred as a result of imported cases entering Malaysia from China and Singapore, while subsequent waves occurred as a result of mass gathering events and introduction of new variants of concern [7,38]. With regard to disease transmissibility, the first wave reported much lower disease transmissibility (R_0_ = 0.9) compared to the second (highest R_t_ = 3.4) and third (highest R_t_ = 1.72) [7]. In addition, the outbreak control measures transitioned from containment (first wave) to mitigation strategies during subsequent COVID-19 waves in Malaysia [38]. Containment measures such as early detection, isolation/quarantine and extensive contact tracing were effective in controlling the outbreak during the first wave as there were low case numbers, the majority of which were imported cases that were identified, tested, and isolated early. In addition, the extensive contact tracing performed enabled the quarantine of close contacts and detection of additional cases, which subsequently prevented local transmissions. During subsequent waves of COVID-19, mitigation strategies were instituted, as there were more cases with higher disease transmissibility that were attributed to mass gathering events [7,38] and the introduction of new variants of concern.

Nevertheless, this paper provides evidence that instituting early detection, isolation, and contact tracing measures, especially during the initial stage of an outbreak (when cases are low in numbers), did reduce disease transmission and subsequently contained the outbreak early on during the first wave.

The limitations of this study include the small number of COVID-19 cases during the first wave. We acknowledge that a smaller data set could affect the estimation of the disease transmissibility (R_0_) during the first wave. Nevertheless, as COVID-19 is a novel disease, it was important to describe the epidemiological characteristics of the first wave despite the small data set to improve our understanding on the initial evolution and progression of the COVID-19 pandemic in Malaysia, as there has been no published studies on the first wave in Malaysia. Despite the small number of case during the first wave, this study was able to describe the initial epidemiological characteristic of the first wave (in terms of case/close contacts/spatial spread/disease transmissibility) and provide an explanation as to the lower number of cases during and early termination of the first wave. 

## 5. Conclusions

This study concludes that all cases were promptly investigated and epidemiological links were successfully established for majority of cases. Similarly, all close contacts of cases (*n* = 368) were successfully traced, identified, tested, isolated and quarantined. As a result of these interventions (i.e., as early case detection, active screening, extensive contact tracing, testing and prompt isolation/quarantine), the outbreak was contained and controlled during the first wave. In addition, the SEIR model developed in this study using several parameters that were estimated based on the study data (i.e., the average number of contacts per day per case, the proportion of close contact traced per day and the mean daily rate at which infectious cases are isolated) estimated a R_0_ less than 1.0, which further supports the decreasing disease dynamics and early termination of the outbreak. As a result, there was an 11-day gap (free of cases) between the first and second wave, which indicates that the first wave was not linked to the second wave.

## Figures and Tables

**Table 1:** Parameters and the respective values used in the SEIR model for the first COVID-19 wave in Malaysia.

Parameter	Description	Value	Source
*n*	Total human population in Malaysia	32,000,000	Department of Statistics Malaysia, 2019 [14]
1/φ	Incubation period	5.2 days	Backer et al., 2020 [15]
β	Force of infection	0.052	Gill et al., 2020 [11]
1/γ	Infectious period	3.6 days	Read et al., 2020 [16]
ε	Death rate due to COVID-19	0	Parameter estimated in this study
ζ	The average number of contacts per day per case	4.82	Parameter estimated in this study
*q*	The proportion of close contact traced per day	0.23	Gill et al., 2020 [11]
1/θ	The duration of quarantine	14	Gill et al., 2020 [11]
κ	The proportion of exposed persons who performed effective precautions	0.05	Gill et al., 2020 [11]
δ	The mean daily rate at which infectious cases are isolated	0.3	Parameter estimated in this study

**Table 2:** Characteristics of the first wave of cases of COVID-19 in Malaysia.

Characteristics	*n* (%)
Gender	
Female	12 (54.5)
Male	10 (45.5)
Age mean (SD) (year)	40.7 (21.6)
1–9 (Child)	3 (13.6)
10–18 (Adolescent)	1 (4.5)
>18–64 (Adult)	15 (68.1)
≥65 (Older adult)	3 (13.6)
Nationality	
Chinese	15 (68.2)
Malaysian	6 (27.3)
United States	1 (4.5)
Type of case	
Imported	20 (90.9)
Local transmission	2 (9.1)
Symptomatic	
No	2 (9.1)
Yes	20 (90.9)
Comorbidity	
No	21 (95.5)
Yes	1 (4.5)
Symptoms (*n* = 20)	
Fever	17 (85.0)
Cough	14 (70.0)
Sore throat	4 (20.0)
Myalgia	3 (15.0)
Headache	4 (20.0)
Running nose	4 (20.0)
Lethargy	1 (5.0)
Shortness of breath	2 (10.0)
Diarrhoea	3 (15.0)
Chest pain	1 (5.0)
Haemoptysis	1 (5.0)
Nasal congestion	1 (5.0)
Onset of symptoms *	
Upon arrival in Malaysia	4 (20.0)
After arrival and before/at admission	13 (65.0)
After admission	1 (5.0)
No symptoms	2 (10.0)
Duration from arrival to admission (Min-Max, Mean (SD)) *	
Overall	0–19, 6.8 (6.8)
Symptomatic at arrival (*n* = 4)	0–10, 3.3 (4.6)
Asymptomatic at arrival (*n* = 16)	0–19, 7.6 (7.1)
Admitting hospital	
Hospital Sungai Buloh, Selangor	8 (36.4)
Hospital Permai, Johor	4 (18.2)
Hospital Kuala Lumpur	4 (18.2)
Hospital Tuanku Jaafar, Negeri Sembilan	2 (9.1)
Hospital Sultanah Maliha, Langkawi	2 (9.1)
Hospital Sultanah Bahiyah, Kedah	2 (9.1)
Case severity	
Mild	19 (86.4)
Severe	3 (13.6)
Treatment	
Symptomatic	18 (82.0)
Antiviral	4 (18.0)
Duration of hospitalization	4–23, 14.9 (5.8)

Note: * Imported cases only (*n* = 20), local cases with no travel history (Cases #13 and #17) excluded.

**Table 3:** Characteristics of close contacts of first wave COVID-19 cases in Malaysia.

Characteristic	*n* (%)
Age (*n* = 259)	
1–9 (Child)	18 (6.9)
10–18 (Adolescent)	18 (6.9)
19–64 (Adult)	205 (79.2)
≥65 (Older adult)	18 (6.9)
Gender (*n* = 329)	
Male	140 (42.6)
Female	189 (57.4)
Nationality (*n* = 350)	
Malaysia	274 (78.3)
China	53 (15.1)
Singapore	13 (3.7)
France	2 (0.6)
US	2 (0.6)
India	1 (0.3)
Indonesia	1 (0.3)
Japan	1 (0.3)
Korea	1 (0.3)
New Zealand	1 (0.3)
Taiwan	1 (0.3)
Type of contact (*n* = 368)	
Health care worker	106 (29.3)
Contacts on flight *	94 (26.0)
Family	53 (14.6)
Work colleague	7 (1.9)
Other contacts **	102 (28.2)

Note: * Includes cabin crew, members of travel group, other passengers on same flight. ** Includes friends, neighbours, attendees of alumni gathering and public transport driver.

## Data Availability

Sourced from the Crisis Response and Preparedness Centre (CPRC), Ministry of Health (MOH) Malaysia.
